# A Master-Slave Binary Grey Wolf Optimizer for Optimal Feature Selection in Biomedical Data Classification

**DOI:** 10.1155/2021/5556941

**Published:** 2021-10-12

**Authors:** Enock Momanyi, Davies Segera

**Affiliations:** Department of Electrical and Information Engineering, University of Nairobi, Nairobi 30197, Kenya

## Abstract

A new master-slave binary grey wolf optimizer (MSBGWO) is introduced. A master-slave learning scheme is introduced to the grey wolf optimizer (GWO) to improve its ability to explore and get better solutions in a search space. Five high-dimensional biomedical datasets are used to test the ability of MSBGWO in feature selection. The experimental results of MSBGWO are superior in terms of classification accuracy, precision, recall, *F*-measure, and number of features selected when compared to those of the binary grey wolf optimizer version 2 (BGWO2), binary genetic algorithm (BGA), binary particle swarm optimization (BPSO), differential evolution (DE) algorithm, and sine-cosine algorithm (SCA).

## 1. Introduction

A number of datasets especially of biomedical nature are high dimensional. This means that they have a high number of features per sample. Most of these features can be described as either redundant or irrelevant and introduce noise which affects the performance of a classifier used in medical diagnosis. It is therefore important to apply dimensionality reduction methods that will select the most informative subset of features. Feature selection is one such method [1].

Depending on the search strategy, the feature selection methods can be categorized as wrapper, filter, and embedded methods. Wrapper methods have an underlying learning algorithm used to evaluate the quality of selected features. Filter methods are efficient in terms of execution time and are independent of any learning algorithm. The embedded methods include both the wrapper and filter methods [2, 3]. This paper focuses on the wrapper method for feature selection.

Since feature selection is an NP-hard problem, there are two traditional methods used to solve it. The methods are the exact method and metaheuristics [2]. Exact methods are time consuming as they have to consider each and every subset, and this becomes computationally expensive as the search space increases. Metaheuristic algorithms which are nature inspired are generally preferred. They are able to find optimal solutions without traversing the entire search space of a given problem. Examples of metaheuristic algorithms that have been used in feature selection include the genetic algorithm (GA) [4], particle swarm optimization (PSO) [5], ant colony optimization (ACO) [6], salp swarm algorithm (SSA) [7], krill nerd algorithm (KNA) [8], dragonfly algorithm [9], grasshopper optimization algorithm [10], whale optimization algorithm [11], firefly algorithm [12], ant lion optimizer [13], emperor penguins algorithm [14], and sine-cosine algorithm [15].

The grey wolf optimizer (GWO) developed by Mirjalili et al. [16] in 2014 mimics the social and hunting behavior of the grey wolves in nature. The GWO is quite popular for its excellent search capability [17] and has the advantage of having few control parameters and fast convergence rate. It has been used in a number of fields including unmanned combat aerial path planning [18], medical diagnosis [19], economic dispatch [20], intrusion detection [21], EMG signal classification [22], and solving engineering problems [23]. However, in the presence of a large search space, the GWO is vulnerable to getting trapped in the local optima.

Researchers have suggested various methods to help it improve its global search capability. In [17], variable weights are used in determining the position of a wolf and an exponential control parameter was also introduced, and the experimental results showed its dominance over the GWO, ALO, PSO, and bat algorithms. In [19], the GWO is hybridized with the genetic algorithm (GA), and using the kernel extreme learning machine (KELM), it outperformed the GA and GWO in the performance metrics on the Parkinson and breast cancer datasets. Also in [24], the particle swarm optimization (PSO) and GWO are combined, and the results are superior compared to those of other algorithms. The concept of competition is introduced among the population of wolves in [22] and outperformed the binary grey wolf optimizer, binary genetic algorithm, and binary particle swarm optimization in classification. The Powell local optimization method is introduced to the GWO for clustering analysis and compared to some evolutionary algorithms; the results were better on the benchmark functions and datasets considered [25].

Despite these improvements, no method has been able to exhaustively find the optimal solution when it comes to feature selection. In an effort to improve the exploration ability of GWO to escape the local optimum, this paper proposes a master-slave binary grey wolf optimizer (MSBGWO) algorithm. This proposed methodology alters the position of the wolves during exploration and exploitation and ensures diversification of the solutions to be considered.

The main contributions of this paper are as follows:

- The MSBGWO introduces a master-slave learning mechanism that makes the bottom half of wolves in terms of fitness to learn from the top half in a sequential manner.
- The proposed MSGW is applied to five highly dimensional datasets. The experimental results show that it is able to select the fewest set of features and obtain higher classification accuracy

The rest of the paper is arranged as follows.  Section 2 gives the background information of GWO.  Section 3 presents the proposed master-slave binary grey wolf optimizer.  Section 4 outlines the experimental design.  Section 5 details the experimental results and discussion, and finally, Section 6 draws the conclusion.

## 2. Grey Wolf Optimizer

**Figure 1:**
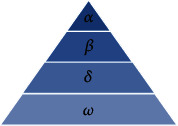
Social hierarchy.

It is part of the swarm intelligence family. It mimics the social and hunting behavior of the grey wolf as stated before [16]. Grey wolves generally move in groups of 5-12 members. The social structure shown in Figure 1 comprises alphas that are the top most, betas that rank just below the alphas, omegas that lie at the bottom, and finally deltas that are neither omegas nor the top two.

The hunting behavior involves finding prey, encirclement and harassment of the prey to restrict its movement, and then finally attacking the prey.

The process of encircling the prey can be modeled mathematically as in the equations below:

(1) $$\begin{array}{c} \overset{⟶}{X}\left(t+1\right)=\overset{⟶}{X}\left(t\right)-\overset{⟶}{A}.\overset{⟶}{D}, \end{array}$$

(2) $$\begin{array}{c} \overset{⟶}{D}=\overset{⟶}{C}.\overset{⟶}{X_{p}}\;\left(t\right)-\overset{⟶}{X}\left(t\right), \end{array}$$

where $\overset{⟶}{X_{w}}$ and $\overset{⟶}{X_{p}}$ are positional vectors of the wolf and prey, respectively, *t* represents the iteration, and $\overset{⟶}{A}$ and $\overset{⟶}{C}$ are vector coefficients.

The vectors $\overset{⟶}{A}\;$ and $\overset{⟶}{C}$ can be determined as follows:

(3) $$\begin{array}{c} \overset{⟶}{A}=2\overset{⟶}{a}.\overset{⟶}{r1}-\overset{⟶}{a}, \end{array}$$

(4) $$\begin{array}{c} \overset{⟶}{C}=2.\overset{⟶}{r2}, \end{array}$$

where **r**1 and **r**2 are random numbers uniformly distributed between [0, 1] and *a* is the encircling coefficient which is linearly decreased from 2 to 0 as the iterations increase according to the equation below:

(5) $$\begin{array}{c} a=2-2\left(\frac{t}{T}\right), \end{array}$$

where *t* is the number of iterations and *T* is the maximum number of iterations.

Hunting is usually led by the alpha. Beta and delta can occasionally participate in hunting. Since we have no idea of the position of the optimum prey, we assume that alpha, beta, and delta have a better knowledge of the position and thus can lead the rest of the pack. Mathematically, this is achieved by selecting the top three fittest solutions which are then used to update the other positional vectors of the grey wolves.

The new position of the wolf is updated as follows:

(6) $$\begin{array}{c} \overset{⟶}{X}\left(t+1\right)=\frac{\overset{⟶}{X_{1}}+\overset{⟶}{X_{2}\;}+\overset{⟶}{X_{3}}}{3}, \end{array}$$

where $\overset{⟶}{X_{1}}$, $\overset{⟶}{X_{2}}$, and $\overset{⟶}{\;X_{3}}$ are calculated as follows:

(7) $$\begin{array}{c} \overset{⟶}{X_{1}}=\overset{⟶}{X_{\alpha }}-\overset{⟶}{A_{1}}.\overset{⟶}{D_{\alpha }}, \end{array}$$

(8) $$\begin{array}{c} \overset{⟶}{X_{2}}=\overset{⟶}{X_{\beta }}-\overset{⟶}{A_{2}}.\overset{⟶}{D_{\beta }}, \end{array}$$

(9) $$\begin{array}{c} \overset{⟶}{\;X_{3}}=\overset{⟶}{X_{\delta }}-\overset{⟶}{A_{3}}.\overset{⟶}{D_{\delta }}, \end{array}$$

where $\overset{⟶}{X_{\alpha }}$, $\overset{⟶}{X_{\beta }}$, and $\overset{⟶}{X_{\delta }}$ are the position of alpha, beta, and delta at iteration *t*, respectively. $\overset{⟶}{D_{\alpha }}$, $\overset{⟶}{D_{\beta }}$, and $\overset{⟶}{D_{\delta }}$ are defined in the equations below:

(10) $$\begin{array}{c} \;\overset{⟶}{D_{\alpha }}=\overset{⟶}{C_{1}}.\overset{⟶}{X_{\alpha }}-\overset{⟶}{X}, \end{array}$$

(11) $$\begin{array}{c} \overset{⟶}{D_{\beta }}=\overset{⟶}{C_{1}}.\overset{⟶}{X_{\beta }}-\overset{⟶}{X}, \end{array}$$

(12) $$\begin{array}{c} \overset{⟶}{D_{\delta }}=\overset{⟶}{C_{1}}.\overset{⟶}{X_{\delta }}-\overset{⟶}{X}. \end{array}$$

For continuous optimization problems, the GWO is used. However, feature selection is a binary optimization problem; thus, the GWO is modified to a binary version which has already been developed [4].

## 3. Master-Slave Binary Grey Wolf Optimizer (MSBGWO)

In each generation or iteration of the grey wolf optimizer, the best three solutions are used in updating the position of each wolf. The omega wolves constitute a larger percentage of the population and have lower fitness in relation with the alpha, beta, and delta wolves. By repositioning the weaker wolves in a guided approach, we can improve the diversification ability of GWO in search of better solutions.

A master-slave learning scheme is hereby introduced. In each generation, the wolves are sorted in ascending order of fitness. The top half are then termed master wolves, and the remaining half become slave wolves. Each slave wolf is assigned a master wolf from whom they will learn.

The slaves will learn from the master using the following equations:

(13) $$\begin{array}{c} D_{L}=\omega .\left|C_{4}.\left(X_{M}-X_{S}\right)\right|, \end{array}$$

where *D*_*L*_ is the fraction of distance between a master and slave wolf, *ω* *ϵ* [0 1] is the learning coefficient, *C*_4_ is determined by equation (4), *X*_*M*_ is a master wolf, *X*_*S*_ is a slave wolf, and *S* and *M* are evaluated using equation (14).

For a population of *N* wolves,

(14) $$\begin{array}{c} S=M+\frac{N}{2}\;M=1,2,3\cdots . \end{array}$$

Following the principles of equation, the new continuous position of the slave wolves is calculated as follows:

(15) $$\begin{array}{c} X_{n}=X_{M}-A_{4}.D_{L}, \end{array}$$

where *X*_*n*_ is the continuous solution and *A*_4_ is determined as in equation (3).

Since feature selection is a binary problem, the continuous solutions are forced to be binary [26].

(16) $$\begin{array}{c} X_{Sd}\left(t+1\right)=\left\{\begin{array}{cc} 1, & \text{if }S\left(X_{n}\right)>\mathrm{rand},\\ 0, & otherwise, \end{array}\right. \end{array}$$

where *X*_*Sd*_ is the new binary solution for a slave wolf in dimension *d*, rand *ϵ* [0 1], and *S* is a sigmoid function given by

(17) $$\begin{array}{c} S\left(x\right)=\frac{1}{1+\exp\left(-10*\left(x-0.5\right)\right)}. \end{array}$$

Equation (16) is also applied when the positions of all wolves are updated but rand is now set as 0.5.

The slave wolves can now be integrated with the master grey wolves in the population and can now move to the next generation.

To increase the number of iterations in the exploration stage, the nonlinear control parameter *a* adopted in [17] is used in place of

(18) $$\begin{array}{c} a=2\left(1-\frac{t^{2}}{T^{2}}\right). \end{array}$$

**Figure 2:**
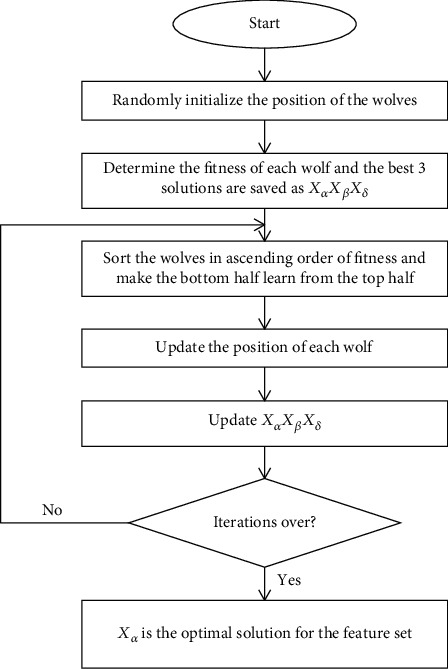
Flowchart for the proposed MSBGWO.

The pseudocode of MSBGWO is presented in Algorithm 1, and the flowchart is shown in Figure 2.

## 4. Experimental Design

### 4.1. Datasets

A total of five high-dimensional biomedical datasets obtained from [4] were used for validation. Each dataset has two labels. The datasets are shown in Table 1.

### 4.2. Evaluation Measures

Using the 10-fold cross-validation method, the prediction models are evaluated based on accuracy, precision, recall, and *F*-measure:

(19) $$\begin{array}{c} Accuracy=\frac{TP+TN}{TP+FP+TN+FN}, \end{array}$$

(20) $$\begin{array}{c} Precision=\frac{TP}{TP+FP}, \end{array}$$

(21) $$\begin{array}{c} Recall=\frac{TP}{TP+FN}, \end{array}$$

(22) $$\begin{array}{c} F‐measure=\frac{2*Precision*Recall}{Precision+Recall}, \end{array}$$

where true positive (TP) is the number of positive samples that are correctly identified as positive, true negative (TN) is the number of negative samples correctly identified as negative, false positive (FP) is the number of negative samples identified as positive, and false negative (FN) is the number of positive samples identified as negative.

Each algorithm is run *k* times, and the results are averaged as follows:

(23) $$\begin{array}{c} AvgAcc=\frac{1}{k}\overset{k}{\underset{i=1}{\sum }}{Accuracy}_{i}, \end{array}$$

(24) $$\begin{array}{c} AvgPre=\frac{1}{k}\overset{k}{\underset{i=1}{\sum }}{Precision}_{i}, \end{array}$$

(25) $$\begin{array}{c} AvgRec=\frac{1}{k}\overset{k}{\underset{i=1}{\sum }}{Recall}_{i}, \end{array}$$

(26) $$\begin{array}{c} AvgF=\frac{1}{k}\overset{k}{\underset{i=1}{\sum }}F‐measure_{i}, \end{array}$$

(27) $$\begin{array}{c} AvgSF=\frac{1}{k}\;\overset{k}{\underset{i=1}{\sum }}Selected-features_{i}. \end{array}$$

### 4.3. Fitness Function

Feature selection is a biobjective problem concerned with minimizing misclassification errors and minimizing the number of features selected.

Thus, the fitness function is determined by the following equation which is from [27].

(28) $$\begin{array}{c} fit=\left(1-\alpha \right).\frac{S}{D}-\alpha .AvgAcc, \end{array}$$

where AvgAcc is the average accuracy determined by the KNN classifier, *S* is the number of selected features, *D* is the total number of features, and *α* is set to 0.8 in this paper.

### 4.4. Parameter Setting

The performance of the proposed MSGWO is compared to that of the binary grey wolf optimizer version 2 (BGWO2), binary genetic algorithm (BGA), binary particle swarm optimization (BPSO), differential evolution (DE) algorithm, and sine-cosine algorithm (SCA). The parameter values for the algorithms are listed in Table 2.

The value of *ω* was selected as 0.1 after values ranging 0.1–1 were considered.

The population size is set to 10 in each of the algorithms, and the number of iterations is set at 100. To complete the wrapper-based approach, a KNN classifier with Euclidean distance, *k* = 5, is also used. A KNN classifier performs optimally when dealing with normalized data, and therefore, all datasets were normalized in the preprocessing step.

Each algorithm is run 10 times on an Intel® Core™ i5 CPU M 520 @ 2.40 GHz to provide a good measure of the results. The implementation is in MATLAB.

## 5. Experimental Results and Discussion

Experimental results of the proposed MSGWO were compared to those of BGWO2, BGA, BPSO, DE, and SCA. The classification accuracy, precision, sensitivity, and *F*-measure over 10 runs using 10-fold CV have been averaged using equations (23)–(27) in Section 4.2 to provide the final results. Box plots have also been used to probe the variations.

### 5.1. Colon Cancer Dataset Results

**Figure 3:**
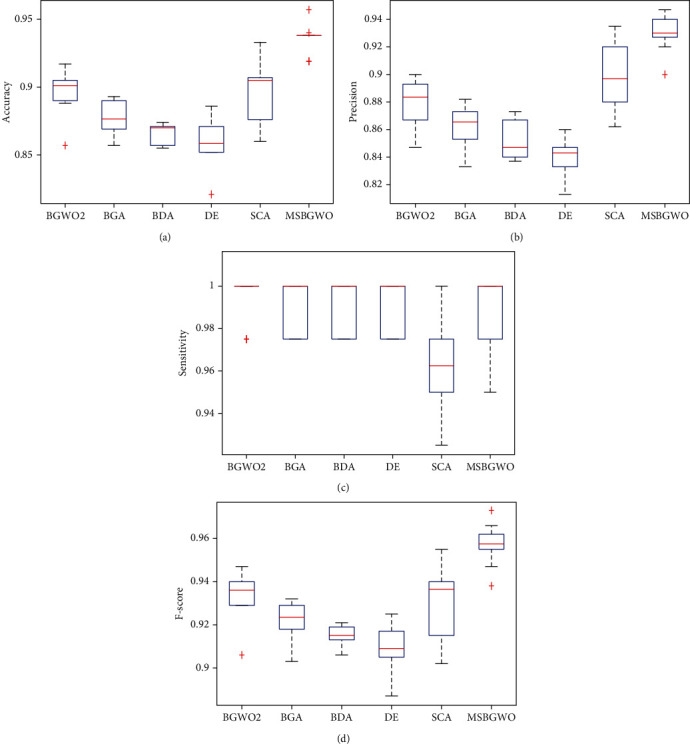
Box plots on accuracy, precision, sensitivity, and *F*-measure on the colon cancer dataset.

Table 3 presents the detailed results on the colon cancer dataset. In the table, we see that the proposed MSBGWO was able to achieve the highest classification accuracy of 0.957. The minimum accuracy of 0.919 when MSBGWO was used to select features was higher than that of BGWO2, BGA, BPSO, and DE. It was only lower than that of SCA. The average classification accuracy, average precision, and average *F*-measure were also the best for MSBGWO among the algorithms considered having selected the fewest features. However, BGWO2 was able to achieve the highest average sensitivity. BPSO was more stable as it had the lowest standard deviation in the average classification accuracy (lower than that of MSBGWO). The box plot in Figure 3 also shows that the median values for accuracy, precision, sensitivity, and *F*-measure are way above those of the other algorithms. The overall superiority of MSBGWO can be attributed to its ability to diversify its solution and minimize being trapped in the local optima.

### 5.2. Central Nervous System (CNS)

**Figure 4:**
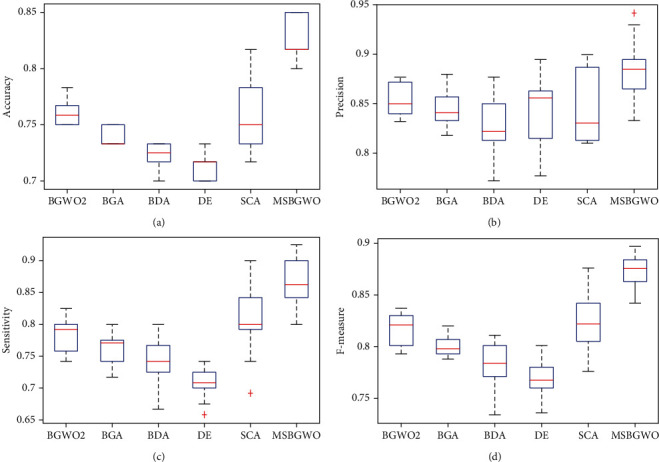
Box plots on accuracy, precision, sensitivity, and *F*-measure on the CNS dataset.

With the values represented in bold in Table 4, we see that using MSBGWO, the values of accuracy, precision, sensitivity, and *F*-measure were the best compared to those of the other algorithms. It also selected the fewest features.  Figure 4 shows the box plots and the median values for accuracy, precision, sensitivity, and *F*-measure which are above those of other algorithms considered. This shows that MSBGWO was more explorative in the search space than the other algorithms ensuring that it selected the most informative features.

### 5.3. Leukemia Dataset Results

**Figure 5:**
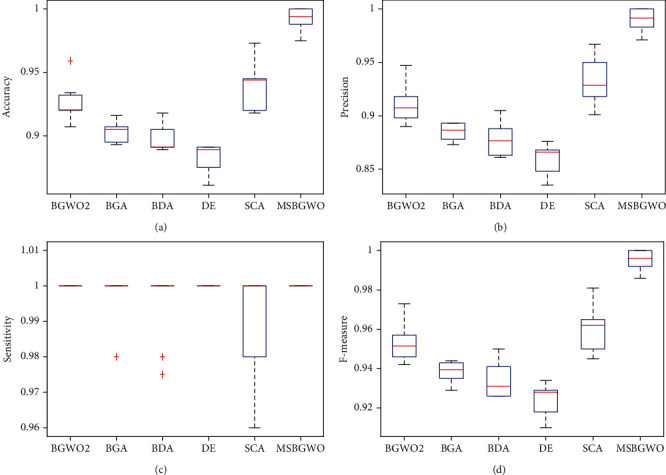
Box plots on accuracy, precision, sensitivity, and *F*-measure on the leukemia dataset.

In Table 5, it is noted that a sensitivity of 100% was obtained when BGWO2, DE, and MSGWO were used for feature selection. We again see that MSBGWO selected the fewest number of features in comparison with the other algorithms. The average values of classification accuracy, precision, and *F*-measure using the proposed MSBGWO are also the best among the algorithms. In fact, the minimum accuracy obtained using MSBGWO betters the maximum achieved by the other algorithms. In the pictorial representation using a box plot in Figure 5, we note that the median values are way superior. The ability of MSBGWO to avoid the local optima by increasing the exploration in the less fit wolves is validated by its superior results.

### 5.4. DLBCL Dataset Results

**Figure 6:**
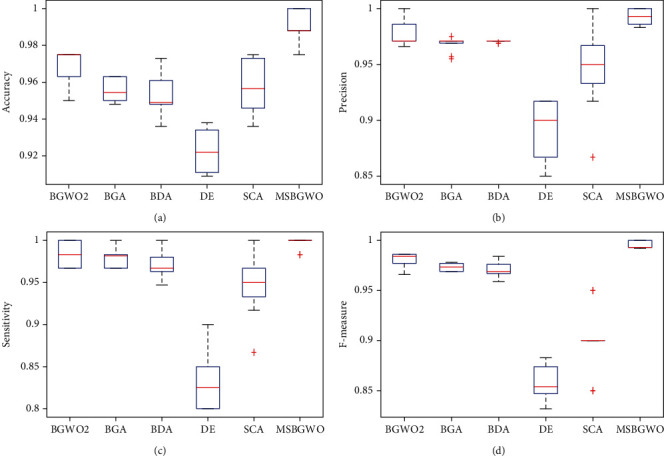
Box plots on accuracy, precision, sensitivity, and *F*-measure on the DLBCL dataset.

In Table 6, we note that using MSBGWO, we attained the highest classification accuracy. The minimum classification accuracy for MSBGWO also matched the maximum classification accuracies for BGWO2 and SCA. The average values for accuracy, precision, sensitivity, and *F*-measure for MSBGWO were highest among the algorithms. This is shown as well in the box plots in Figure 6 where the median values obtained using MSGWO are superior.

### 5.5. Ovarian Cancer Results

**Figure 7:**
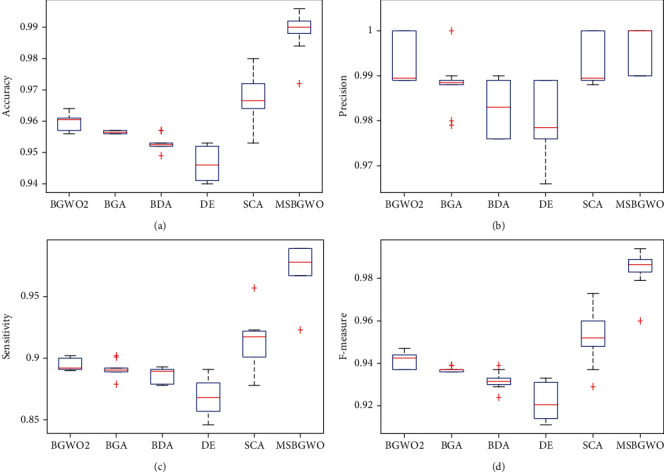
Box plots on accuracy, precision, sensitivity, and *F*-measure on the ovarian cancer dataset.

From Table 7, the MSBGWO proves to be superior as it selected the fewest features on average and had the highest classification accuracy and its minimum classification accuracy was not bettered by maximum accuracy of the remaining algorithms. Average values for precision, sensitivity, and *F*-measure were highest among the algorithms considered. The box plots in Figure 7 also represent this as we see that the median values for those metrics are highest.

### 5.6. Wilcoxon Rank Sum Test

We use this nonparametric statistical test to evaluate if the median values for MSGBWO against BGWO2, BGA, BPSO, DE, and SCA are equal at 5% significance level as shown in Table 8. The null hypothesis is that the median values of two samples will be equal, and the alternative hypothesis is unequal median values. *h* = 0 represents the null hypothesis, and *h* = 1 rejects the null hypothesis.

In summary, we see that the proposed MSBGWO selected the fewest features which proved to be most informative as the accuracy, precision, sensitivity, and *F*-measure were better than those of BGWO2, BGA, BPSO, DE, and SCA in the datasets in Table 1. This demonstrates the superiority of the algorithm when it comes to feature selection, and the modification of GWO helped in diversification.

## 6. Conclusion

A master-slave binary grey wolf optimizer is proposed in this paper. A master-slave learning scheme is introduced to improve the exploration ability of the grey wolf optimizer. Five biomedical datasets are used to test the strength of the proposed MSBGWO. The experimental results show that the proposed algorithm outperforms the BGWO2, BGA, BPSO, DE, and SCA in the performance metrics considered in this paper. In future work, the proposed algorithm can be used in noncontinuous optimization problems. From the results, we see that BGA was the most stable; thus, hybridizing BGA and MSGBWO should be a consideration.

## Figures and Tables

**Algorithm 1:**
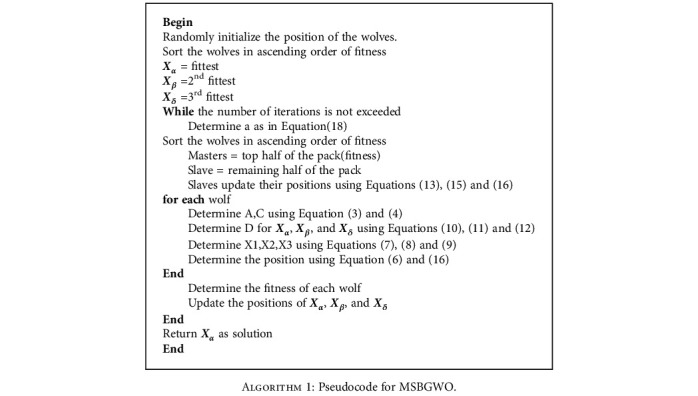
Pseudocode for MSBGWO.

**Table 1:** Datasets.

Dataset	Instances	Features
Colon cancer	2000	62
Central nervous system (CNS)	7129	60
DLBCL	7129	77
Leukemia	7129	72
Ovarian cancer	15154	253

**Table 2:** Parameter values for each algorithm.

Algorithm	Parameters
MSBGWO	*ω* = 0.1
BGA	MR = 0.01, CR = 0.8
BPSO	C1 = 1, C2 = 2, *V*_max_ = 6, *W*_max_ = 0.9, *W*_min_ = 0.4
DE	CR = 0.9, *F* = 0.5
SCA	Alpha = 2

**Table 3:** Experimental results of the algorithms on the colon cancer dataset.

Algorithm	Accuracy			AvgSF	AvgPre	AvgRec	AvgF
	Max	Min	AvgAcc				
BGWO2	0.917	0.857	0.896 ± 0.016	447.1	0.881	0.995	0.934
BGA	0.893	0.857	0.877 ± 0.013	980.9	0.861	0.993	0.922
BPSO	0.874	0.855	0.866 ± 0.008	993.9	0.851	0.990	0.915
DE	0.886	0.821	0.859 ± 0.016	1217.2	0.840	0.993	0.910
SCA	0.933	0.860	0.898 ± 0.021	88.6	0.899	0.960	0.932
MSBGWO	0.957	0.919	0.936 ± 0.011	70	0.930	0.988	0.958

**Table 4:** Experimental results of the algorithms on the CNS dataset.

Algorithm	Accuracy			AvgSF	AvgPre	AvgRec	AvgF
	Max	Min	AvgAcc				
BGWO2	0.783	0.750	0.760 ± 0.012	1648.2	0.855	0.782	0.816
BGA	0.750	0.733	0.740 ± 0.009	3525.5	0.845	0.763	0.801
BPSO	0.733	0.700	0.723 ± 0.011	3518.9	0.827	0.741	0.781
DE	0.733	0.700	0.712 ± 0.011	4251.3	0.843	0.708	0.769
SCA	0.817	0.717	0.760 ± 0.030	458.1	0.844	0.804	0.822
MSBGWO	**0.850**	**0.800**	0.825 ± 0.020	**290.9**	**0.885**	**0.863**	**0.873**

**Table 5:** Experimental results of the algorithms on the leukemia dataset.

Algorithm	Accuracy			AvgSF	AvgPre	AvgRec	AvgF
	Max	Min	AvgAcc				
BGWO2	0.959	0.907	0.925 ± 0.015	1330.8	0.910	1.000	0.953
BGA	0.916	0.893	0.903 ± 0.007	3516.1	0.885	0.998	0.938
BPSO	0.918	0.889	0.897 ± 0.010	3509.9	0.878	0.996	0.933
DE	0.891	0.861	0.884 ± 0.010	4051.0	0.860	1.000	0.925
SCA	0.971	0.918	0.940 ± 0.017	244.7	0.934	0.990	0.961
MSBGWO	1.000	0.975	0.991 ± 0.010	98.2	0.989	1.000	0.995

**Table 6:** Experimental results of the algorithms on the DLBCL dataset.

Algorithm	Accuracy			AvgSF	AvgPre	AvgRec	AvgF
	Max	Min	AvgAcc				
BGWO2	0.975	0.955	0.969 ± 0.009	1470.8	0.978	0.983	0.981
BGA	0.963	0.948	0.956 ± 0.006	3541.3	0.968	0.978	0.973
BPSO	0.973	0.936	0.951 ± 0.011	3532.0	0.971	0.969	0.970
DE	0.938	0.909	0.923 ± 0.010	3999.3	0.895	0.835	0.860
SCA	0.975	0.936	0.957 ± 0.015	359.6	0.945	0.900	0.917
MSBGWO	1.000	0.975	0.992 ± 0.008	182.3	0.993	0.997	0.996

**Table 7:** Experimental results of the algorithms on the ovarian cancer dataset.

Algorithm	Accuracy			AvgSF	AvgPre	AvgRec	AvgF
	Max	Min	AvgAcc				
BGWO2	0.964	0.956	0.960 ± 0.003	2706.6	0.994	0.895	0.942
BGA	0.957	0.956	0.957 ± 0.001	7457.6	0.988	0.891	0.937
BPSO	0.957	0.949	0.953 ± 0.002	7501.6	0.983	0.886	0.932
DE	0.953	0.940	0.946 ± 0.005	7777.5	0.981	0.869	0.921
SCA	0.980	0.953	0.966 ± 0.007	422.4	0.993	0.913	0.951
MSBGWO	0.996	0.972	0.988 ± 0.007	167.9	0.996	0.973	0.984

**Table 8:** Wilcoxon rank sum test at 5% significance level.

Dataset	Wilcoxon rank sum test	BGWO2 vs. MSBGWO	BGA vs. MSBGWO	BPSO vs. MSBGWO	DE vs. MSBGWO	SCA vs. MSBGWO
Colon cancer	*p* value	1.4590*e* − 04	1.4677*e* − 04	1.4332*e* − 04	1.4764*e* − 04	4.8358*e* − 04
	*h* value	1	1	1	1	1
	*z* value	3.7979	3.7965	3.8024	3.7950	3.4897
Central nervous system	*p* value	1.4077*e* − 04	1.2855*e* − 04	1.4077*e* − 04	1.4077*e* − 04	6.5823*e* − 04
	*h* value	1	1	1	1	1
	*z* value	3.8068	3.8292	3.8068	3.8068	3.4064
Leukemia	*p* value	1.5385*e* − 04	1.5028*e* − 04	1.3993*e* − 04	1.4504*e* − 04	1.5385*e* − 04
	*h* value	1	1	1	1	1
	*z* value	3.7848	3.7906	3.8083	3.7994	3.7848
DLBCL	*p* value	2.7222*e* − 04	1.4077*e* − 04	1.4851*e* − 04	1.5116*e* − 04	2.0168*e* − 04
	*h* value	1	1	1	1	1
	*z* value	3.6404	3.8068	3.7935	3.7892	3.7169
Ovarian cancer	*p* value	1.6025*e* − 04	1.3334*e* − 04	1.5475*e* − 04	1.6305*e* − 04	2.9692*e* − 04
	*h* value	1	1	1	1	1
	*z* value	3.7746	3.8202	3.7833	3.7703	3.6180

## Data Availability

Data is available from the corresponding author upon request.

## References

[1] Sinha A., Hripcsak G., Markatou M. (2009). Large datasets in biomedicine: a discussion of salient analytic issues. *Journal of the American Medical Informatics Association*.

[2] Adi S., Aldasht M. (2018). Parallel evolutionary algorithms for feature selection in high dimensional datasets. *International Journal of Computer Science and Information Security (IJCSIS)*.

[3] Li J., Cheng K., Wang S. (2018). Feature Selection. *ACM Computing Surveys (CSUR)*.

[4] Zhu Z., Ong Y.-S., Dash M. (2007). Markov blanket-embedded genetic algorithm for gene selection. *Pattern Recognition*.

[5] N N., Vashishtha J. (2016). Particle swarm optimization based feature selection. *International Journal of Computer Applications*.

[6] Kanan H. R., Faez K., Taheri S. (2007). Feature selection using ant colony optimization (ACO): a new method and comparative study in the application of face recognition system. *Advances in Data Mining. Theoretical Aspects and Applications*.

[7] Ibrahim H. T., Mazher W. J., Ucan O. N., Bayat O. (2017). Feature selection using salp swarm algorithm for real biomedical datasets. *IJCSNS International Journal of Computer Science and Network Security*.

[8] Abualigah L. M. Q. (2019). *Feature Selection and Enhanced Krill Herd Algorithm for Text Document Clustering*.

[9] Mafarja M. M., Eleyan D., Jaber I., Hammouri A., Mirjalili S. Binary dragonfly algorithm for feature selection.

[10] Ibrahim H. T., Mazher W. J., Ucan O. N., Bayat O. (2019). A grasshopper optimizer approach for feature selection and optimizing SVM parameters utilizing real biomedical data sets. *Neural Computing and Applications*.

[11] Hussien A. G., Hassanien A. E., Houssein E. H., Bhattacharyya S., Amin M., Bhattacharyya S., Mukherjee A., Bhaumik H., Das S., Yoshida K. (2019). S-shaped binary whale optimization algorithm for feature selection. *Recent Trends in Signal and Image Processing*.

[12] Emary E., Zawbaa H. M., Ghany K. K. A., Hassanien A. E., Parv B. Firefly optimization algorithm for feature selection.

[13] Mafarja M. M., Mirjalili S. (2019). Hybrid binary ant lion optimizer with rough set and approximate entropy reducts for feature selection. *Soft Computing*.

[14] Baliarsingh S. K., Vipsita S., Muhammad K., Bakshi S. (2019). Analysis of high-dimensional biomedical data using an evolutionary multi- objective emperor penguin optimizer. *Swarm and Evolutionary Computation*.

[15] Abualigah L., Diabat A. (2021). Advances in sine cosine algorithm: a comprehensive survey. *Artificial Intelligence Review*.

[16] Mirjalili S., Mirjalili S. M., Lewis A. (2014). Grey wolf optimizer. *Advances in Engineering Software*.

[17] Gao Z.-M., Zhao J. (2019). An improved grey wolf optimization algorithm with variable weights. *Computational Intelligence and Neuroscience*.

[18] Zhang S., Zhou Y., Li Z., Pan W. (2016). Grey wolf optimizer for unmanned combat aerial vehicle path planning. *Advances in Engineering Software*.

[19] Li Q., Chen H., Huang H. (2017). An enhanced grey wolf optimization based feature selection wrapped kernel extreme learning machine for medical diagnosis. *Computational and Mathematical Methods in Medicine*.

[20] Pradhan M., Roy P. K., Pal T. (2018). Oppositional based grey wolf optimization algorithm for economic dispatch problem of power system. *Ain Shams Engineering Journal*.

[21] Almomani O. (2020). A feature selection model for network intrusion detection system based on PSO, GWO, FFA and GA algorithms. *Symmetry*.

[22] Too J., Abdullah A., Mohd Saad N., Mohd Ali N., Tee W. (2018). A new competitive binary grey wolf optimizer to solve the feature selection problem in EMG signals classification. *Computers*.

[23] Nadimi-Shahraki M. H., Taghian S., Mirjalili S. (2021). An improved grey wolf optimizer for solving engineering problems. *Expert Systems with Applications*.

[24] al-Tashi Q., Abdul Kadir S. J., Rais H., Mirjalili S., Alhussian H. (2019). Binary optimization using hybrid grey wolf optimization for feature selection. *IEEE Access*.

[25] Zhang S., Zhou Y. (2015). Grey wolf optimizer based on Powell local optimization method for clustering analysis. *Discrete Dynamics in Nature and Society*.

[26] Emary E., Zawbaa H. M., Hassanien A. E. (2016). Binary grey wolf optimization approaches for feature selection. *Neurocomputing*.

[27] Salesi S., Cosma G. A novel extended binary cuckoo search algorithm for feature selection.

